# LPS Challenge Regulates Gene Expression and Tissue Localization of a *Ciona intestinalis* Gene through an Alternative Polyadenylation Mechanism

**DOI:** 10.1371/journal.pone.0063235

**Published:** 2013-04-30

**Authors:** Aiti Vizzini, Angela Bonura, Daniela Parrinello, Maria Antonietta Sanfratello, Valeria Longo, Paolo Colombo

**Affiliations:** 1 Dipartimento di Scienze e Tecnologie Biologiche, Chimiche e Farmaceutiche, Palermo, Italy; 2 Istituto di Biomedicina ed Immunologia Molecolare “Alberto Monroy” del Consiglio Nazionale delle Ricerche, Palermo, Italy; UMDNJ-New Jersey Medical School, United States of America

## Abstract

A subtractive hybridization strategy for the identification of differentially expressed genes was performed between LPS-challenged and naive *Ciona intestinalis.* This strategy allowed the characterization of two transcripts (*Ci*8short and *Ci*8long) generated by the use of two Alternative Polyadenylation sites. The *Ci*8long transcript contains a protein domain with relevant homology to several components of the Receptor Transporting Protein (RTP) family not present in the *Ci*8short mRNA. By means of Real Time PCR and Northern Blot, the *Ci*8short and *Ci*8long transcripts showed a different pattern of gene expression with the *Ci*8short mRNA being strongly activated after LPS injection in the pharynx. *In situ* hybridization analysis demonstrated that the activation of the APA site also influenced the tissue localization of the *Ci*8short transcript. This analysis showed that the *Ci*8long mRNA was expressed in hemocytes meanwhile the *Ci*8short mRNA was highly transcribed also in vessel endothelial cells and in the epithelium of pharynx. These findings demonstrated that regulation of gene expression based on different polyadenylation sites is an ancestral powerful strategy influencing both the level of expression and tissue distribution of alternative transcripts.

## Introduction

Alternative Polyadenylation (APA) has recently gained attention as a major player influencing the dynamics of gene regulation [1].

Usually, mature 3′ ends of almost all eukaryotic mRNAs are created by a two-step reaction that involves an endonucleolytic cleavage of the pre-mRNA, followed by synthesis of a polyadenylate tail onto the upstream cleavage product. Polyadenylation influences many aspects of mRNA metabolism: transcription termination by RNAP II, mRNA stability, mRNA export to the cytoplasm and the efficiency of translation are all dependent on 3′ processing.

In recent years it has become increasingly evident that APA regulates gene expression [2]. In some cases the alternative poly(A)^+^ sites are located in internal introns/exons regions (Coding Region Alternative PolyAdenylation CR-APA) leading to different protein isoforms. In other cases, APA sites are all located in the 3′untranslated region (UTR-APA), resulting in transcripts with 3′ UTRs of different length but encoding the same protein. In this way, CR-APA can qualitatively affect the gene expression by producing distinct protein isoforms, whereas UTR-APA quantitatively affects the expression.

Ascidians (subphylum Tunicata) are chordate invertebrates whose immune system relies only on innate immunity including inflammatory humoral and cellular responses [3]–[5]. Due to the knowledge of the *Ciona intestinalis* genome [6], this ascidian has become a model to study the evolution of immune related genes [7]. In particular, previous research has shown that pharynx and hemocytes responses can be challenged by LPS inoculation through the body wall, therefore this experimental setting represents a well-established model to examine innate immunity gene expression. Previously published papers have described the inflammatory response and the immune role of *C. intestinalis* pharynx. In this respect, pharynx epithelia and hemocytes (mainly compartment/morula cells) express immune related genes (coding type IX collagen-like [8], TNFα-like [9], [10], CAP-like [11], MBL-like [12], and galectin-like proteins [13]) upregulated by lipopolysaccharide (LPS). The pharynx occupies an extensive part of the adult body. It consists of two epithelial monolayers perforated by dorso-ventrally aligned rows of elongated elliptical, ciliated stigmata [14] enclosed in a mesh of vessels (also called transversal and longitudinal bars), where the hemolymph, containing abundant mature and immature hemocytes, flows. Hemopoietic nodules are associated with the bar epithelia that can be stimulated by mitogens [15], [16]. In addition, in this organ, a C3-like protein gene is upregulated by LPS [17] suggesting the activation of a lectin-dependent complement-like system [18], while the activation of the proPO-system and an increased release of lectins with opsonic property have been shown [19], [20].

In the present paper, a subtractive hybridization strategy for selective amplification of differentially expressed sequences showed that LPS challenge can induce an alternative polyadenylation mechanism in *C. intestinalis*. The LPS induced model correlates with the up-regulation and differential tissue localization of a novel gene.

## Materials and Methods

### Tunicates and LPS injection

Ascidians were collected from Sciacca Harbour (Sicily, Italy), a non-protected area in the Mediterranean sea, maintained in tanks with aerated sea water at 15°C and fed every second day with a marine invertebrate diet coraliquid (Sera Heinsberg, Germany) according to local guidelines. The work described in this study did not involve endangered or protected species. No specific permits were required for the described field studies.

Lipopolysaccharide (LPS-*Escherichia coli* 055:B5, LPS, Sigma-Aldrich, Germany) solution was prepared in sterile marine solution (12 mM CaCl_2_ X 6H_2_O, 11 mM KCl, 26 mM MgCl_2_ X 6H_2_O, 43 mM TrisHCl, 0.4 M NaCl, pH 8.0). Ascidians were injected into the tunic tissue at the median body region with: marine solution (sham ascidians) and LPS solution (100 µg LPS in 100 µl marine solution per animal). Untreated and sham ascidians were used as controls.

### Total RNA extraction and poly(A)^+^ purification

Ascidian pharynx fragments (200 mg), excised at various times (from 1 to 72 hours), were immediately soaked in RNA later Tissue collection (Ambion, Austin, TX), and stored at −80°C. Total RNA extraction was performed by using an RNAqueous^TM^-Midi Kit purification system (Ambion, Austin, TX).

Poly(A)^+^ RNA was prepared from control and injected animals (1 hour) using Illustra™ mRNA Purification Kit (GE Healthcare, UK) according to the manufacturer's instructions.

### Subtractive hybridization and screening of the cDNA library

Subtractive hybridization was performed using the PCR-Select™ cDNA Subtraction Kit (Clontech Laboratories, USA) according to the manufacturer's instructions. This strategy is based on a PCR-based method for selective amplification of differentially expressed sequences allowing the isolation of transcript from activated tissues. Briefly, 2 µg of poly(A)^+^ RNA from non-injected (driver) and injected (tester) animals (1 h p.i. of LPS) were retro-transcribed. The tester and driver cDNAs were digested with the restriction enzyme Rsa I to yield blunt ends. The tester cDNA was then subdivided into two parts and each was ligated with a different cDNA adaptor (ADAPTOR1: 5′-CTAATACGACTCACTATAGGGCTCGAGCGGCCGCCCGGGCAGGT-3′; ADAPTOR 2: 5′-CTAATACGACTCACTATAGGGCAGCGTGGTCGCGGCCGAGGT-3′).The ends of the adaptor do not contain a phosphate group, so only one strand of each adaptor attaches to the 5′ ends of the cDNA. Then two hybridizations were performed. In the first run, an excess of driver was added to each sample of tester. The samples were then heat denatured and allowed to anneal. In the second run of hybridization, the two primary hybridization samples were mixed together without denaturing to allow the subtracted single strand tester cDNAs to re-associate. These new hybrids were molecules with different ends, which correspond to the sequences of the two adaptors. After filling in the ends by DNA polymerase, the differentially expressed sequences display different annealing sites for the nested primers on their 5′ and 3′ ends. The entire population of molecules is then subjected to PCR to amplify the desired differentially expressed sequences using the following primers (Nested PCR Primer 1 5′-TCGAGCGGCCGCCCGGGCAGGT-3′; Nested PCR Primer 2 5′-AGCGTGGTCGCGGCCGAGGT-3′) and PCR conditions (94°C for 30′′, 68°C for 30′′, 72°C for 1,5′; 12 cycles). Screening of the library was performed hybridizing the subtracted library with P^32^ labeled probes synthesized as first-strand cDNA from tester and driver. Clones corresponding to differentially expressed mRNAs will hybridize only with the tester probe, and not with the driver probe. P^32^ labeled colonies were grown and plasmid DNA extracted.

### Cloning and sequences analysis

**Figure 1:**
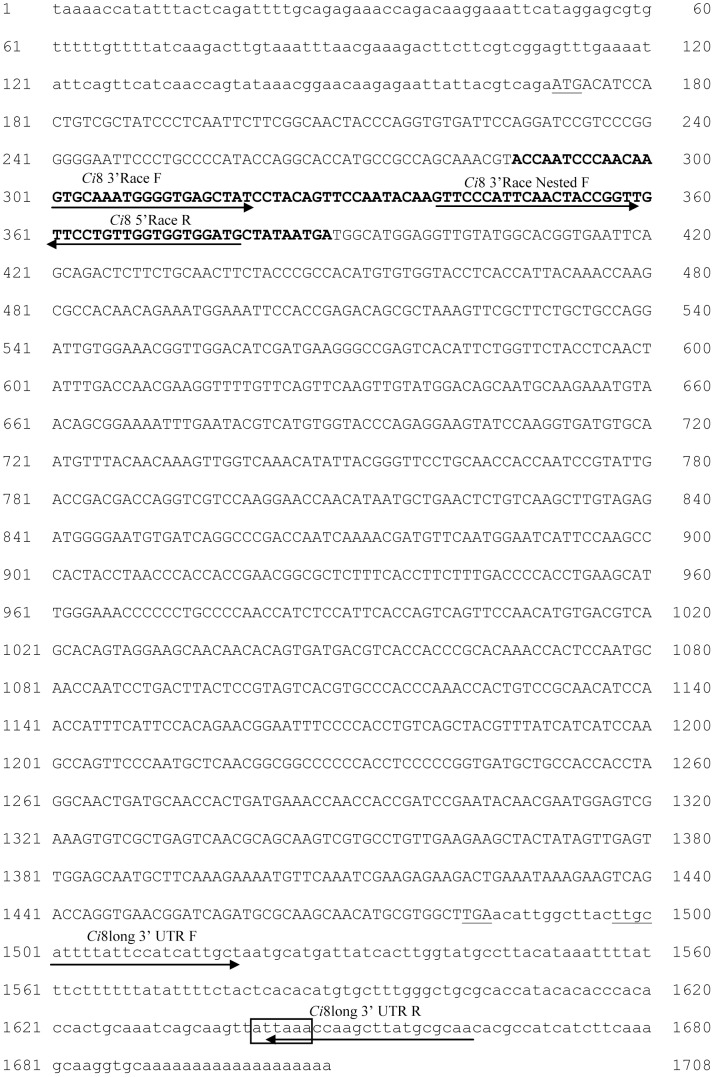
Nucleotide sequence of the full length *Ci*8long cDNA: 5′ and 3′ UTR regions are described in lower case letters; the coding region were in upper case letters; the first ATG and the STOP codon were underlined; sequence in bold displays the 102 bp fragment identified by Subtractive Hybridization. Box shows the canonical *C.intestinalis* polyadenylation site. The arrows indicate the oligonucleotides used for cloning procedures, Real time PCR and ISH assay.

**Figure 2:**
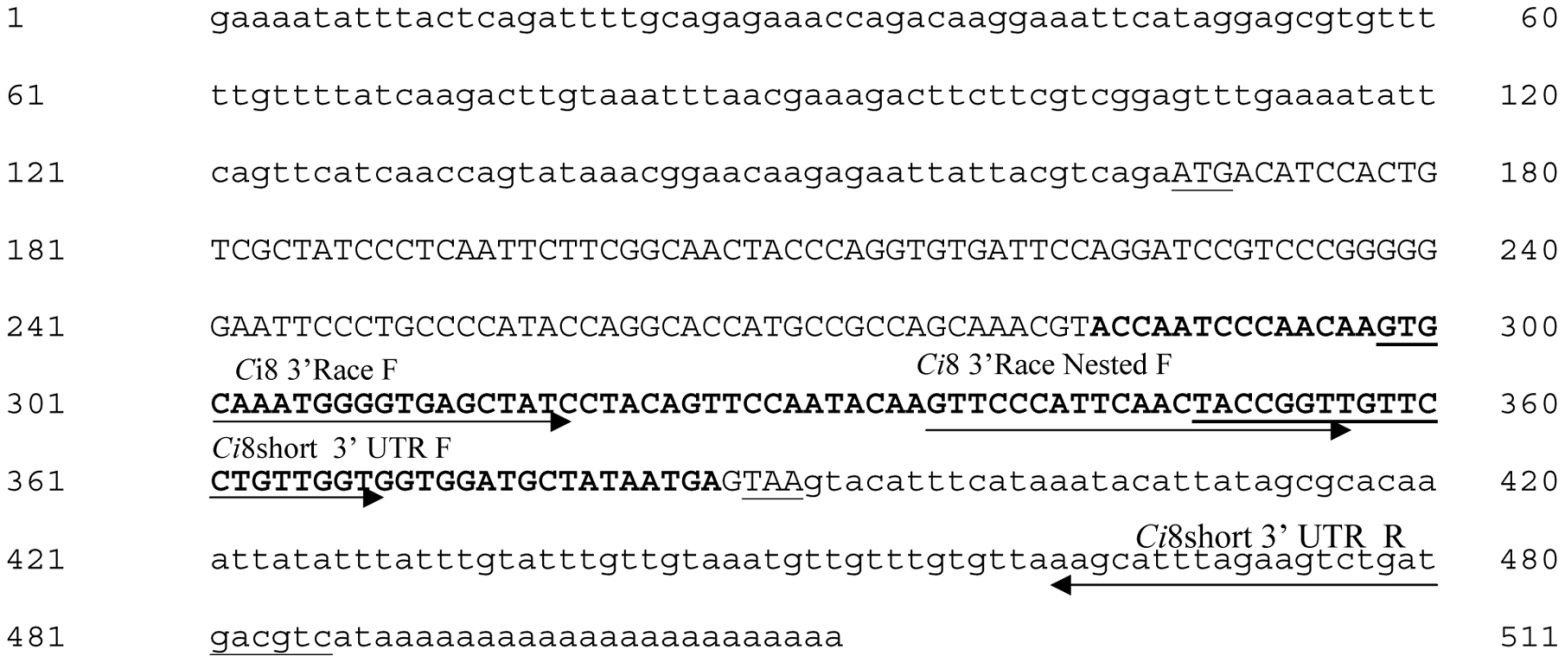
Nucleotide sequence of the full length *Ci*8short cDNA: 5′ and 3′ UTR regions are described in lower case letters; the coding region were in upper case letters; the first ATG and the STOP codon were underlined; sequence in bold display the 102 bp fragment identified by Subtractive Hybridization. The arrows indicate the oligonucleotide used for cloning procedures, Real time PCR and ISH assay.

Differentially expressed cDNA was cloned in the pCR4-TOPO vector (Invitrogen, USA) and sequenced. Sequence analysis identified a cDNA fragment of 102 nucleotides. Similarity searches performed using the FASTA algorithm (http://www.ebi.ac.uk/Tools/fasta/) showed a relevant homology to some EST clones from mature adult *Ciona intestinalis* animal (data not shown). The full length sequence of the cDNA clone was obtained by using the GeneRacer^TM^ kit (Invitrogen, USA). The kit ensures the amplification of only full length transcript via elimination of truncated messages from the amplification process. 5′ RACE was performed by PCR (94°C 1 min, 52°C 1 min, 72°C 1 min for 30 cycles) using the Ci8 5′Race R specific oligonucleotide (5′-CATCCACCACCAACAGGAA-3′) (see Figure 1 for details) and the GeneRacer^TM^ 5′-oligonucleotide (5′- CGACTGGAGCACGAGGACACTGA-3′).The 5′ RACE technology has identified only one fragment of 379 bp;

The 3′ RACE was performed using the Ci8 3′Race F specific oligonucleotide (5′-GTGCAAATGGGGTGAGCTAT-3′) and the GeneRacer^TM^3′ oligonucleotide (3′-GCAATGCATCGCATAGCAACTGTCG-5′). PCR products were diluted 1∶100 and re-amplified using the Ci83′Race Nested F specific oligonucleotide (5′-GTTCCCATTCAACTACCGGT-3′) and the GeneRacer^TM^3′ nested oligonucleotide (5′-GTTCCCATTCAACTACCGGTT-3′) (see Figure 1 and 2 for details). By means of 3′RACE we were able to identify two cDNA fragments: a first one of 1370 bp and a second one of 176 bp. DNA fragments were purified and cloned in the pCR4-TOPO vector (Invitrogen, USA) and sequenced. Sequence analysis showed that both fragments contains a common 50 bp region overlapping with the originally isolated 102 bp fragments (see figures 1 and 2 for details).

In order to uniquely identify the 5′ sequences of the two 3′ RACE cDNA fragments, a second step of 5′RACE analysis was performed. The full length longer cDNA was isolated by RT PCR using the *Ci*8long 3′ UTR R oligonucleotide and the GeneRacer^TM^ 5′-oligonucleotide (named *Ci*8long). The full length shorter cDNA was isolated by PCR using the *Ci*8short 3′UTR R and the GeneRacer^TM^ 5′-oligonucleotide (named *Ci*8short). Two fragments of 1662 and 486 bp were isolated, purified and cloned in the pCR4-TOPO vector (Invitrogen, USA).

### Sequence, structural and phylogenetic analysis

Similarity searches were performed using the FASTA program (http://www.ebi.ac.uk/Tools/fasta/). Multiple alignments were accomplished with the Clustal W program [21]. The final sequence alignment was done using CLUSTAL W v.1.81 [21] and the similarity shaded with CLC workbench 6.4. A phylogenetic tree was constructed by the Neighbor-Joining method (NJ) after 1000 bootstrap iterations by using CLC workbench 6.4. The respective GenBank accession numbers were as follows: ACM09027.1 (*Salmo salar* Receptor transporting protein 3), ACO08037.1 (Oncorhynchus *mykiss* Receptor transporting protein 3, XP_693604.4 (*Danio rerio* Receptor transporting protein 2) XP_002405527.1 (*Ixodes scapularis* Receptor transporting protein), AAT70680.1 (*Homo sapiens* Receptor transporting protein 1), AAT70681.1 (*Homo sapiens* Receptor transporting protein 2), NP_113628.1 (*Homo sapiens* Receptor transporting protein 3), AAH13161.1 (*Homo sapiens* Receptor transporting protein 4), AAT70670.1 (*Mus musculus* Receptor transporting protein 1), AAT70671.1 (*Mus musculus* Receptor transporting protein 2), AAT70672.1 (*Mus musculus* Receptor transporting protein 3*),* AAH24872.1 (*Mus musculus* Receptor transporting protein 4), NP_001179185.1 (*Bos taurus* Receptor transporting protein 1), DAA33411.1 (*Bos taurus* Receptor transporting protein 2*),* NP_001069429.1 (*Bos taurus* Receptor transporting protein 4),XP_003358456.1 (*Sus scrofa* Receptor transporting protein 4), XP_002934100.1 (*Xenopus tropicalis* Receptor transporting protein 3), GAA36455.2 (Clonorchis *sinensis* Receptor transporting protein 3) [21].

Structural prediction was performed using the PSIPRED Protein Structure Prediction Server (http://bioinf.cs.ucl.ac.uk/psipred/) and the Predict Protein algorithm (http://www.predictprotein.org/).

### Gene expression analysis

Tissue differential expression of the two mRNAs was studied by Real-Time PCR using the Sybr-Green method. To discriminate the two transcripts specific sets of primers were designed on 3′UTR regions using Custom Primers OligoPerfect^TM^ Designers software (https://tools.invitrogen.com/) and synthesized commercially (Eurofins MWG Operon, Ebersberg, Germany). Real-time PCR analysis was performed using the Applied Biosystems 7500 real-time PCR System. *Ci*8long isoform tissue expression was performed in a 25 µl PCR reaction containing 2 μl cDNA converted from 250 ng of total RNA, 300 nM C*i*8long 3′UTR forward primer (5′-TTGCATTTTATTCCATCATTGC-3′) and *Ci*8long 3′ UTR Reverse primers (5′-TTGCGCATAAGCTTGGTTTA-3′), 300 nM actin forward (5′-TGATGTTGCCGCACTCGTA-3′) and reverse (5′- TCGACAATGGATCCGGT-3′) primers, and 12.5 μl of Power Sybr-Green PCR Master Mix (Life Technologies, Milan, Italy).

*Ci*8short expression was performed in the same PCR conditions with 300 nM *Ci*8short 3′UTR Forward primer(-5′TACCGGTTGTTCCTGTTGGT-3′)and 300 nM *Ci*8short 3′UTR Reverse specific primer (5′-GACGTCATCAGACTTCTAAATGCT-3′).

The 50 cycles of the two-step PCR program consisted of initial polymerase activation for 3 min at 95°C followed by denaturing step at 95°C for 15 sec, and then the annealing/extension was carried out at 60°C for 45 sec when the fluorescent signal was detected. Each set of samples was run three times and each plate contained quadruplicate cDNA samples and negative controls.

The specificity of amplification was tested with real time PCR melting analysis. To obtain sample quantification, the 2^−ΔΔCt^ method was used and the relative changes in gene expression was analysed as described in the Applied Biosystems Use Bulletin N.2 (P/N 4303859). The amount of *Ci*8long and *Ci*8short transcripts from different tissues was normalized to actin in order to compensate for variations in input RNA amounts. Relative *Ci*8long and *Ci8* short expression was determined by dividing the normalized value of the target gene in each tissue by the normalized value obtained from the untreated tissue.

Northern blot analysis was performed as previously described [22]. A nucleotide fragment corresponding to the coding region of the *Ci*8short cDNA (including the common region between the two mRNAs) was labelled with α-CTP32 and the Rediprime II DNA Labeling System (GE Healthcare Life Science, Milan, Italy). Membrane was exposed to a Kodak X-Omat AR X-ray film for 48 hours.

### Pharynx explants preparation and histology

The tunic surface was cleaned and sterilized with ethyl alcohol and pharynx fragments (200 mg) were excised from the injection site of sham and LPS challenge ascidians. For *in situ* hybridization studies, pharynx fragments were fixed in Bouin′s fluid (saturated picric acid:formaldehyde:acetic acid 15∶5∶1) for 24 hours, paraffin embedded, and serially cut at 6 μm (Leica RM2035 microtome, Solms, Germany).

### *In situ* hybridization assay (ISH)

To examine tissue excised from the inflamed body wall, ISH was carried out with digoxigenin-11-UTP-labeled riboprobes (1 µg/ml final concentration). The *Ci*8long probe was generated by PCR amplifying a cDNA fragment of 165 bp covering the 3′untranslated region from nucleotide 1496 to nucleotide 1662 of the isolated cDNA using the *Ci*8long 3′UTR forward oligonucleotide (5′-TTGCATTTTATTCCATCATTGC-3′) and the *Ci*8long 3′UTR reverse oligonucleotides (5′-TTGCGCATAAGCTTGGTTTA-3′) (see Figure 1).

The DNA fragment was cloned in the pCR4-TOPO vector (Invitrogen, USA). The *Ci*8short probe was generated by PCR amplifying a cDNA fragment of 138 bp covering the 3′untranslated region from nucleotide 348 to nucleotide 486 of the isolated cDNA using the *Ci*8 short 3′UTR forward primer (-5′TACCGGTTGTTCCTGTTGGT-3′)and the *Ci*8 short 5′UTR Race Reverse specific oligonucleotide (5′-GACGTCATCAGACTTCTAAATGCT-3′) (see Figure 2).

The digoxigenin-11-UTP-labeled riboprobes was carried out according to manufacturer′s instructions (Roche Diagnostics). The re-hydrated histological sections were digested with proteinase K (10 µg/ml) in PBS for 5 min, washed with PBS-T, and treated for hybridization with 50% formamide, 5X SSC, 50 µg/ml heparin, 500 µg/ml yeast tRNA, and 0.1% Tween 20, at 37°C overnight. After exhaustive washing in PBS-T and 4XSSC (twice for 10 min), the sections were incubated for 1hr with anti-DIG-Fab-AP conjugate (Roche Diagnostics, Milan, Italy) diluted 1∶500 and washed in PBS-T. Finally, the sections were incubated in the 5-bromo-4-chloro-3-indolyl phosphate/nitro blue tetrazolium liquid substrate system (Sigma-Aldrich, Milan, Italy). Colour development was stopped after 30 min at room temperature.

### Statistical methods

Student′s t-test was used to estimate statistical significance. Multiple comparisons were performed with one-way analysis of variance (ANOVA) and different groups were compared by using Tukey′s t-test. Standard deviations were calculated on four experiments. P<0.01 was considered statistically significant.

## Results

### Isolation of the *Ci*8short and *Ci*8long cDNAs generated by alternative APA

**Figure 3:**
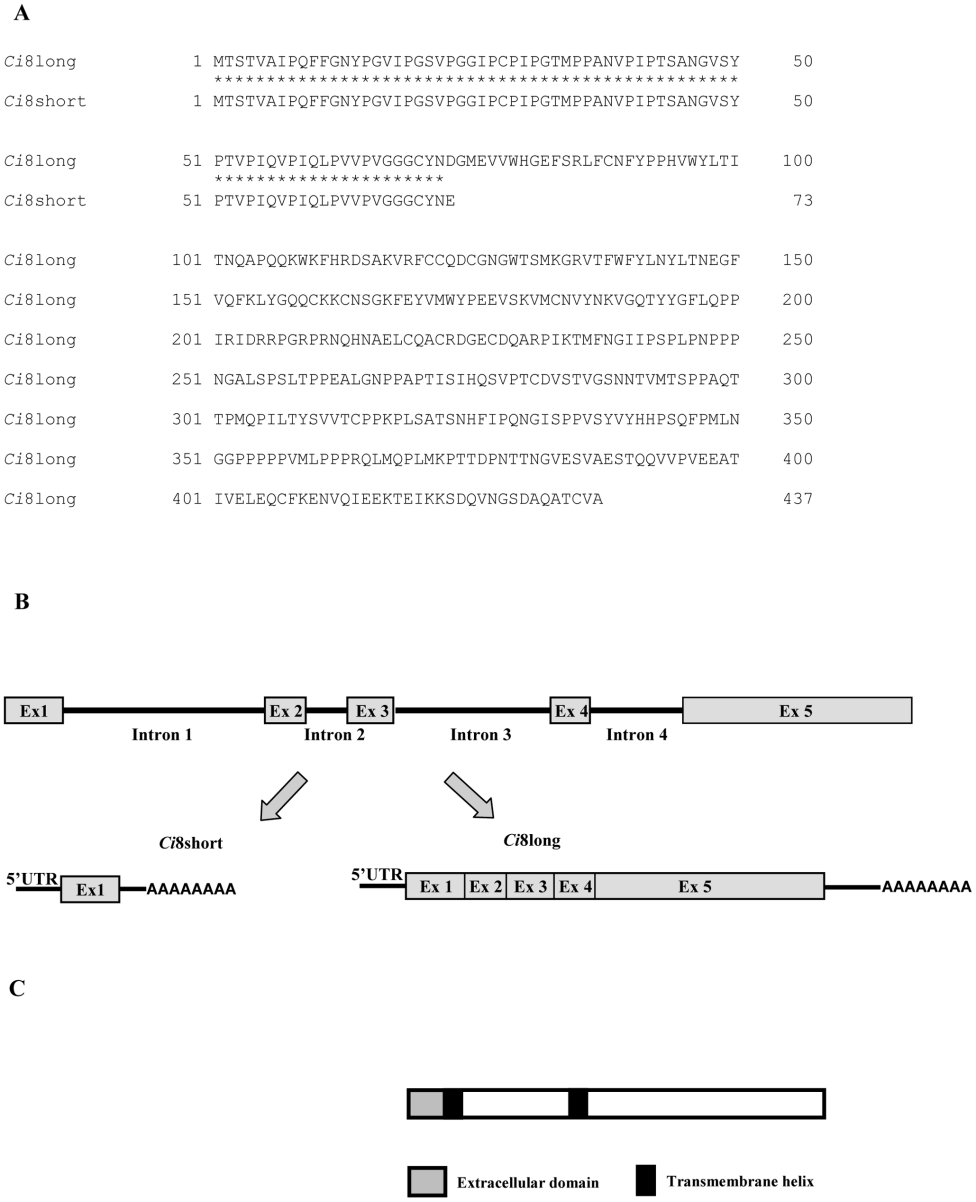
Amino acid comparison and structural analysis of *Ci*8long and *Ci*8short proteins. **Panel A**) Alignment of *Ci*8long and *Ci*8short deduced amino acid sequences. Asterisks indicate amino acid identity. **Panel B**) Schematic representation of *Ciona intestinalis* ENSCING00000009651 gene, *Ci*8long and *Ci*8short transcripts. **Panel C**) Schematic representation of the *in silico* analysis of the Ci8long deduced amino acid sequences.

By means of a PCR-based subtractive hybridization strategy and 5′ and 3′ Gene RACE, two full-length cDNAs were identified from mRNA extracted from the pharynx of *C. intestinalis* after LPS injection. A first cDNA of 1708 nucleotides (named *Ci*8long) showed short 5′ and 3′untranslated regions (170 and 224 nucleotides, respectively) and an open reading frame of 1314 nucleotides coding for a 437 amino acid long protein (putative MW 47973.90 Dalton) (see Figure 1 and Figure 3 panel A for details). A second cDNA, named *Ci*8short, consisted of a 511 nucleotides fragment with 5′ and 3′untranslated regions of 167 and 122 nucleotides, respectively (Figure 2). The *Ci*8short cDNA contains an open reading frame of 219 nucleotides coding for a 73 amino acid long protein (putative MW 7328.54 Dalton) (Figure 3 panel A). Alignments between the *Ci*8long and the *Ci*8short deduced amino acid sequences showed that the *Ci*8short protein represents a shorter form of the *Ci*8long protein (Figure 3 panels A and B).

**Figure 4:**
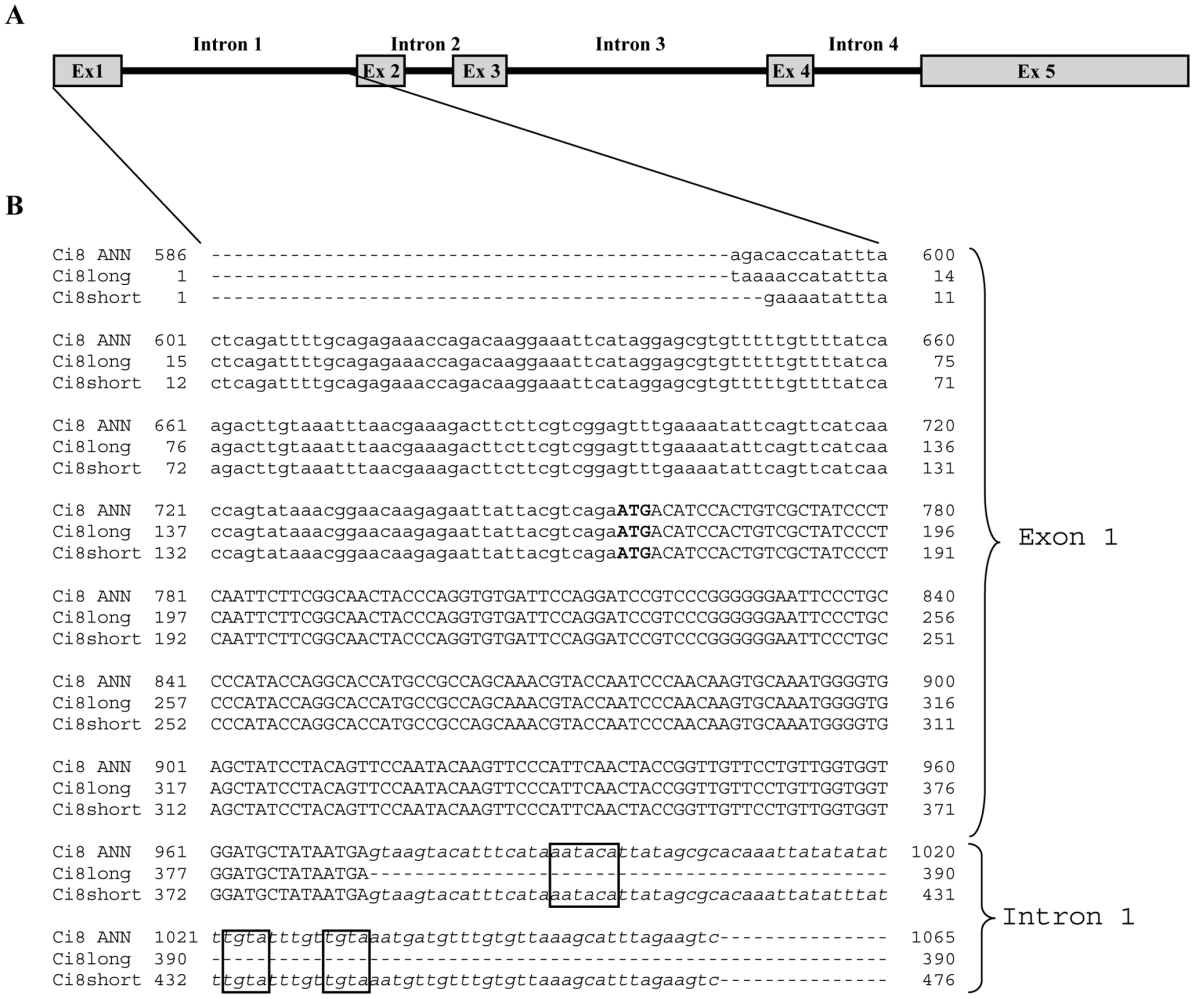
Genomic structure of the *Ci*8long gene. **Panel A**) schematic representation of the *Ciona intestinalis* ENSCING00000009651 gene (Ci8 ANN); **B**) Alignment of the Exon 1 and Intron 1 of *Ciona intestinalis* gene (Ci8 ANN) with the *Ci*8long fragment (1–390 bp) and *Ci*8short full length sequence. 5′ UTR regions were described in lower case letters; the first ATG was highlighted in bold. The *Ci*8short 3′UTR corresponds to the first 91bp of the first intron of the annotated gene (Ci8 ANN). Boxes show the non-canonical *C.intestinalis* “AAUACA” polyadenylation sites and the tetranucleotide sequences”UGUA”.

A search in Ensembl genome browser performed with the *Ci*8long nucleotide sequence identified a five exons and four introns gene (ENSCING00000009651) localized on Chromosome 5: 555,293–559,003. This analysis identified a unique transcript (ENSCINT00000019621) for this gene. Then, a more detailed analysis was performed aligning the nucleotide sequences of the *Ci*8long, the *Ci*8short and the sequence of the annotated transcript (ENSCING00000009621). The *Ci*8long matches with the entire coding sequence of the annotated transcript. A comparison between the *Ci*8short versus the annotated genomic sequence showed that it matches with the 5′untranslated region, the first 218 nucleotides of the coding region (corresponding to the first exon sequence) plus 91 nucleotides lying within the first intron of the gene (Figure 3 Panel B). In this region, we identified a non-canonical polyadenylation site (AAUACA) between nucleotides 402–407. In addition, two conserved tetranucleotides elements (UGUA) were identified in the positions 433–436 and 442–445, respectively (Figure 4).

On the other hand, the *Ci8*long 3′ UTR was analysed for the presence of polyadenylation sites. The *Ci8*long cDNA displays an ATTAAA sequence located between nucleotides 1640 and 1646 which is considered the most frequent variant of the canonical polyadenylation site [23] (Figure 1). In conclusion, *in silico* analysis showed that the 3′ untraslated regions of the two mRNAs differ in the length, sequence and polyadenylation signals.

### *In silico* structural analysis

*In silico* structural analysis of the C*i*8long protein showed two putative transmembrane regions between aa 72–90 and aa 173–192 (Figure 3 panel C). None of these regions were detected in the *Ci*8short deduced sequence. In addition, the residue composition analysis of the *Ci*8short deduced sequence revealed a high percentage of proline and glycine residues (22% and 13%, respectively).

### Phylogenetic analysis of the Ci8long RTP-like domain

**Figure 5:**
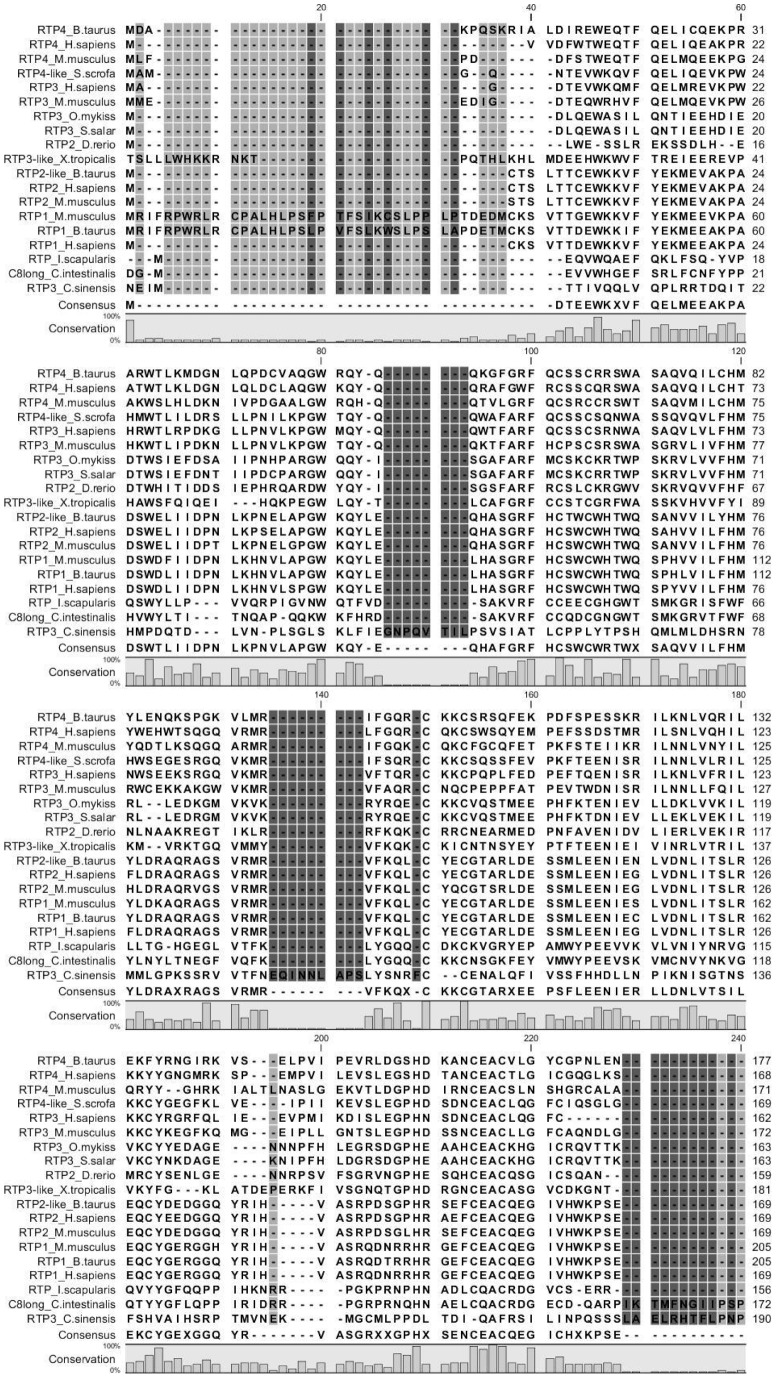
Alignment of *Ci*8long Receptor Transporting Protein domain with Receptor Transporting Protein domain of invertebrate (Ixodes scapularis, Clonorchis sinensis) and vertebrate (Salmo salar, Oncorhynchus mykiss, Danio rerio, Xenopus tropicalis, [RTP1, RTP2 and RTP4 from Bos Taurus], Sus scrofa, [RTP1-4 from Mus musculus], [RTP1-4 from Homo sapiens]). The conservation of amino acid is represented by letter background colour gradients (from black to white).

A FASTA3 search showed that the *Ci8*long transcript contains a protein domain with relevant homology to several components of the Receptor Transporting Protein (RTP) family. This RTP-like domain, absent in the *Ci*8short transcript, displays a high percentage of similarity (SP) and identity (IP) with vertebrate and invertebrate RTPs: 64% SP and 55% IP with *Ixodes scapularis* RTP (arthropod), 46% SP and 27% IP with *Danio rerio* RTP2, 41% SP and 31% IP with *Salmo salar* RTP3, 45% SP and 30% IP with *Oncorhynchu mykiss* RTP3, 41% SP and 25% IP with *Mus musculus* RTP 4, 42% SP and 25% IP with *Bos taurus* RTP4, 41% SP and 21% IP with *Homo sapiens* RTP 4. *Ci*8long Receptor Transporting Protein domain was aligned with Receptor Transporting Protein domain of invertebrate (*Ixodes scapularis, Clonorchis sinensis)* and vertebrate (*Salmo salar, Oncorhynchus mykiss, Danio rerio, Xenopus tropicalis, Bos taurus, Sus scrofa, Mus musculus, Homo sapiens*) (Figure 5).

**Figure 6:**
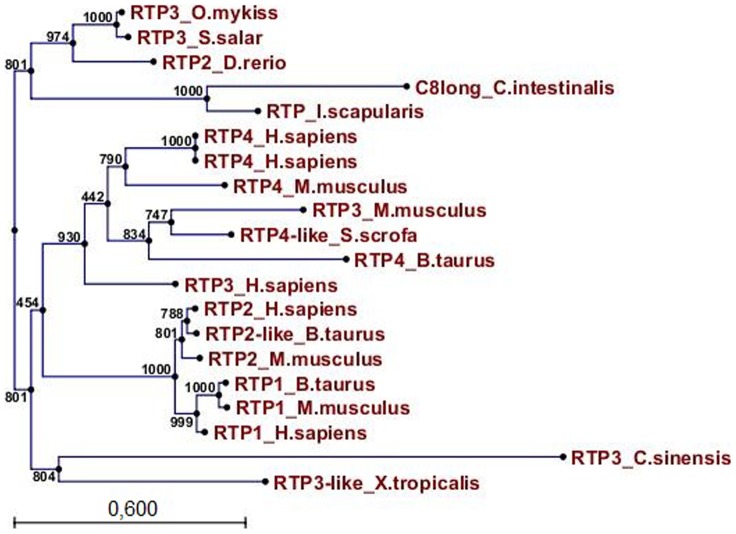
Phylogenetic tree of vertebrate and invertebrate components of Receptor Transporting Protein family. The tree was constructed by the neighbour-joining method and bootstrap analysis. Bootstrap value indicates the percentage of time that the particular node occurred in 1000 trees generated by bootstrapping the sequences. Bar 0.6 (number of amino acid residues substitutions for site).

The phylogenetic tree, constructed by comparing vertebrate and invertebrate components of the RTP family, showed the following main clusters. The first one containing fish RTP2 (*D.rerio)* and RTP3 (*S. salar, O. mykiss*), the C*i*8long and the arthropod RTP sequences (*I. scapularis*). The second one consists of mammal RTPs separated into the RTP3 and RTP4 subgroups (*H. sapiens, M. musculus, B.taurus, S.scrofa*) and the RTP1 and RTP2 subgroups. The third one consists of the amphibian *X. tropicalis* and the flatworm *C.sinensis* RTP 3-like (Figure 6).

### Differential expression of *Ci*8long and *Ci*8short transcripts disclosed by Real Time PCR and Northern blotting

To study the expression pattern of the *Ci*8long and *Ci*8short mRNAs, specific primers were designed within the 3′ UTR of the two cDNAs (see Figure 1 and Figure 2). Quantitative mRNA expression of *Ci*8long and *Ci*8short in naive, sham and LPS challenged ascidians was examined by Real Time PCR analysis. Four naive, sham and LPS-treated ascidians in three distinct experiments were examined at different post-inoculation time points (1, 4, 8, 12, 24, 48, 72 h). The LPS treated ascidians were compared to specimens inoculated with marine solution, and the latter compared to naive ascidians.

**Figure 7:**
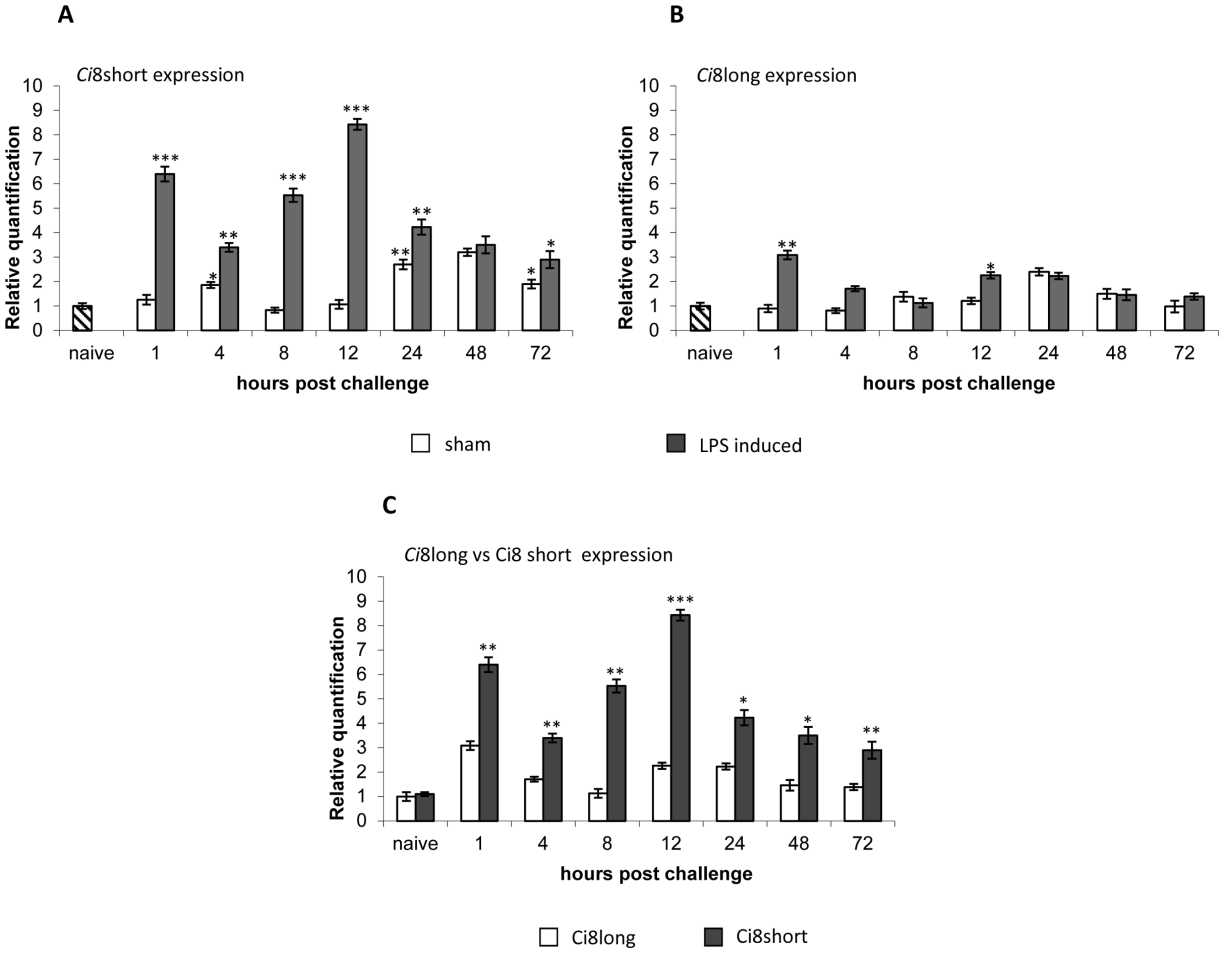
Real-time PCR analysis. Comparison of the *Ci*8short (Panel A) and *Ci*8long (Panel B) gene expression in *Ciona intestinalis* pharynx in LPS-injected, sham and naïve ascidians. Panel C shows the comparison between the *Ci*8short and *Ci*8long expression in LPS-injected animals. *P<0.05, **P<0.01, ***P<0.001.

In the LPS-treated ascidians, *Ci*8short expression, compared to the *Ci*8long one, disclosed a significantly higher RNA level at all time points (P<0.01). In particular, the *Ci*8short expression was enhanced at 1 h and reached a maximum of expression 12 h p.i., then decreased at 72 h p.i. (Figure 7, panel A).

The *Ci*8long mRNA level was slightly enhanced at 1 and 12 h p.i. (Figure 7, panel B). The inoculation procedure (sham ascidians) slightly modulated the expression levels in comparison to the naive specimens (Figure 7, panel A e B).

In addition, Figure 7 panel C shows the comparison of the level of expression of the Ci8short mRNA versus the *Ci8*long one. This assay demonstrates that the number of molecules of the short mRNA is statistically higher in the LPS challenged ascidians at all the time points.

**Figure 8:**
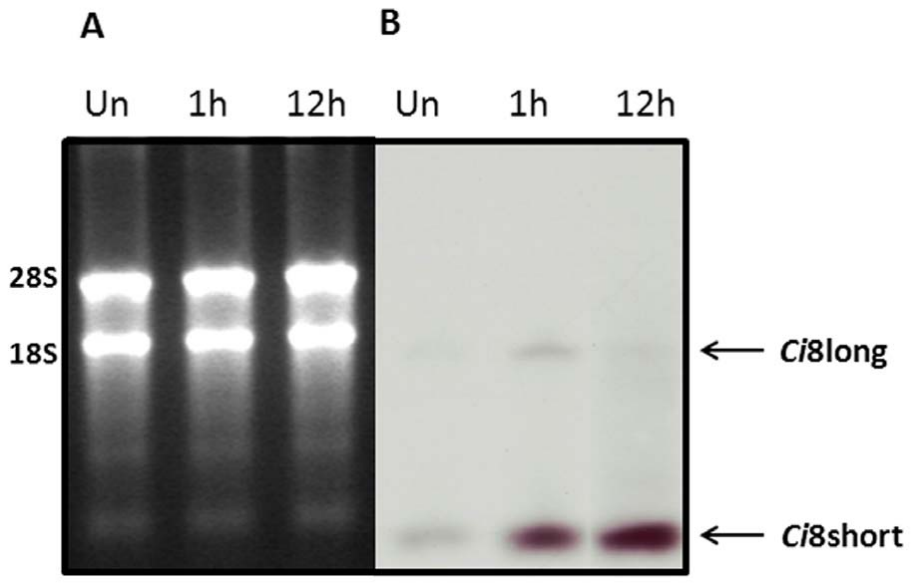
Northern blot analysis. Panel A shows the ethidium bromide staining of the RNA extracted from *Ciona intestinalis* pharynx in naive ascidians (Un) and LPS-injected ascidians (1 h and 12 hours p.i.). Panel B shows the same gel blotted on membrane and hybridized with a P^32^ labelled fragment corresponding to the coding region of the Ci8short cDNA.

The data are in agreement with the Northern blot assay showed inside Figure 8. Total RNA, from pharynx from naive and LPS-challenged ascidians 1 hour and 12 hours p.i., were fractionated and hybridized with a P^32^ labeled probe covering the coding region of the *Ci*8short cDNA. This analysis showed a faint band corresponding to the size of the *Ci*8long mRNA in all the lines and an increasing hybridization signal in LPS-challenged ascidians in a region of about 500 nucleotides corresponding to the size of the *Ci*8short mRNA.

### *In situ* hybridization assay of pharynx

**Figure 9:**
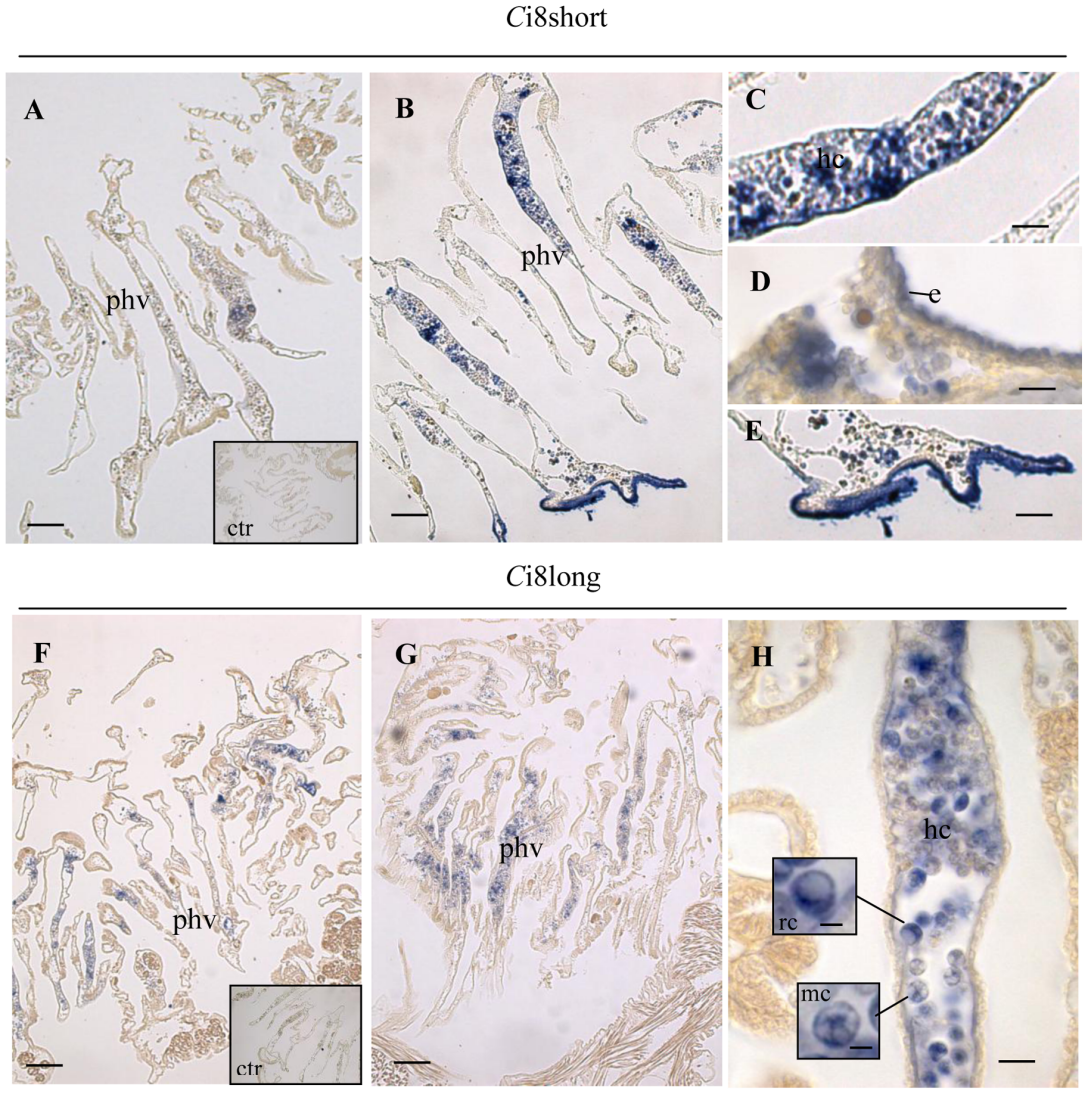
Histological sections of *Ciona intestinalis* pharynx. *In situ* hybridization with the *Ci*8short riboprobe: sham ascidian (Panel A) and ascidian at 12 h after LPS challenge (Panel B). Panels C-E show magnification of vessels and endothelium reported inside Panel B. *In situ* hybridization with the *Ci*8long riboprobe: sham ascidian (Panel F) and ascidian at 12 h after LPS inoculation (Panel G). Panel H shows magnification of a vessel inside panel G. Panel H inserts: signet ring cells (rc) and compartiment\morula cells (mc).Bars size: 50 μm (Panels A, B), 25 µm (Panels C, E), 10 µm (Panels D, H), 100 µm (Panels F, G), 5 μm (Panel H insets); phv: pharynx vessels, ctr: sense strand control hybridization, e: epithelium, hc: hemocyte cluster.

Figure 9 shows histological sections of the pharynx containing hemocytes from sham (panels A and F) and LPS-treated ascidians (panels B,C,D,E,G and H) 12 h p.i.

The *Ci*8short localization in the pharynx from LPS-treated ascidians shows an enhanced gene expression (Figure 9 panels B,C,D and E) when compared to the sham (panel A). In particular, a large part of the vessels appeared to be densely populated with hemocytes expressing the *Ci*8short transcript (Figure 9 panels B,C). The *Ci*8short is manly expressed by endothelial cells (panel d) that appeared to be marked in various regions of the pharynx bars (panel E).

On the contrary, differences in *Ci*8long transcript expression could not be observed between sham (Figure 9, panel F) and LPS treated ascidians (Figure 9, panel G). Figure 9 panel H shows that the *Ci*8long transcript is mainly expressed in compartment/morula and signet ring cells located in tightly packed cluster within the vessel lumen (Figure 9 panel H, insets).

Histological sections treated with the sense strand (negative control) did not display any positive staining.

## Discussion

In recent years it has become evident that APA is an important mechanism in vertebrate and invertebrate organisms to produce different protein isoforms (coding region-APA) or regulate gene expression (UTR-APA). Differential processing at multiple poly(A) sites can be influenced by physiological and pathological conditions such as cell growth, differentiation, development, cancer and stress condition such as inflammation [1].

In this paper, we used a subtractive hybridization strategy on the attempts to identify LPS differentially expressed sequences in the *C.intestinalis* pharynx tissue that has been retained to be the main protagonist of the innate immunity responses. This strategy allowed us the identification of two mRNAs (*Ci*8long and *Ci*8short) derived from the transcription of the (ENSCING00000009651) annotated gene. In particular, LPS was able to weakly modulate the expression of the *Ci*8long transcript and to induce the activation of a LPS-induced APA mechanism responsible for the generation of a shorter mRNA (*Ci*8short). In fact, *in silico* analysis identified a non-canonical polyadenylation site within the first intron of the annotated gene. This region was composed by the hexanucleotide AATACA followed by two short tetranucleotides (TGTA). The latter sequences have been shown to be involved in alternative polyadenylation events in vertebrate binding specific cleavage factors [24].

Sequence analysis showed that the *Ci*8long deduced amino acid sequence displays a protein domain with homology to components of the Receptor Transporting Protein (RTP) family [25]. The RTP family is composed of four members (RTP1-4) who were first identified as partners for mammalian odorant receptors, promoting cell surface expression and leading to functional responses in heterologous cell system. RTP1 and RTP2 are expressed in olfactory neurons and vomeronasal neurons, RTP3 is expressed in liver, lung and testis and RTP4 is expressed in a wide variety of tissues including lymph nodes, peripheral blood leucocytes, spleen and thymus (reviewed in [25]). The mechanism of action of this family of proteins is poorly understood and the existence of several closely related family members with disparate phenotypes suggests a wide role of these proteins.

The phylogenetic analysis supports that the CiRTP-like domain identified in the *Ci*8long RNA is a component of the RTP family while discloses the close relationship of RTPs inside the chordate clade sharing a common ancestor. Moreover, the RTP-like sequence found in the arthropod *I. scapularis* suggests a more ancient progenitor. A similar consideration arises from the phylogenetic branch formed with the amphibian *X. laevis* and the flatworm *C. sinensis* RTP3-like sequences.

We do not know the functional role of the presumptive RTP-like protein as deduced from *Ci*8long cDNA sequence as well as of the presumptive protein encoded by the *Ci*8short sequence. In any case, the short isoform do not contain the RTP domain and do not display any other homolog in the data banks different from the *C.intestinalis* annotated transcript (data not shown).

Furthermore, *in silico* prediction demonstrated that the *Ci*8long derived protein contains two transmembrane regions which are not present in the *Ci*8short protein suggesting that the short isoform may represent an LPS induced secreted form of the constitutively expressed gene.

In fact, it is noteworthy that *Ci*8long is expressed both in naive and LPS-challenged ascidians while the *Ci*8short transcript is significantly enhanced during the inflammatory process.

In particular, as demonstrated by means of Real Time PCR, the *Ci*8short expression profile showed a peak of activation within 1 hour p.i. followed by a second wave of activation at the stage of 12 hours. Interestingly, a similar pattern of activation has been observed for the expression of other LPS-induced components of the *C. intestinalis* inflammatory response [8], [9], [11]–[13]. These data are in agreement with the Northern blot analysis.

Furthermore, the tissue localization of the *Ci*8short and *Ci*8long transcripts showed that LPS inoculation also induced a differential tissue localization of the two mRNAs probably related to the APA mechanism. The *Ci*8long transcript was expressed in some hemocytes of pharynx vessels, whereas the *Ci*8short mRNA appears to be strongly up regulated in compartment/morula and signet ring cells as well as in vessel endothelial cells and epithelium. In this respect, compartment/morula cell types are known to populate inflamed tissues engaged in the expression of immune related genes [8], [9], [11]–[13]. Although precise quantitative data were not derived from the histological observations, the possibility that an increased number of Ci8short positive hemocytes in the vessels as well as positive regions of the endothelium can be related to LPS inoculation is supported by previous published papers [8], [9], [11]–[13].

Finally, the finding that pharynx tissues gene expression can be upregulated by LPS is consistent with evidence on C3-like gene expression [17], and supports the finding that ascidian pharynx is involved in immune-surveillance. This is in accordance with the role of this organ that comes in contact with a large variety of microbes exerting an early recognition of Pathogen-associated molecular patterns.

### Conclusions

This paper reports on the first identification of a CiRTP-like protein and an LPS induced APA mechanism in the invertebrate chordate *Ciona intestinalis*. In this respect, we showed that the mechanism based on different polyadenylation sites is an ancestral powerful strategy of gene regulation interfering with the level of expression and tissue distribution of alternative transcripts.
